# Predicting Depression in Community Dwellers Using a Machine Learning Algorithm

**DOI:** 10.3390/diagnostics11081429

**Published:** 2021-08-07

**Authors:** Seo-Eun Cho, Zong Woo Geem, Kyoung-Sae Na

**Affiliations:** 1 Department of Psychiatry, Gachon University College of Medicine, Gil Medical Center, Incheon 21565, Korea; arztin01@gilhospital.com; 2 College of IT Convergence, Gachon University, Seongnam 13120, Korea; zwgeem@gmail.com

**Keywords:** mental health, depression, LASSO, logistic regression, machine learning

## Abstract

Depression is one of the leading causes of disability worldwide. Given the socioeconomic burden of depression, appropriate depression screening for community dwellers is necessary. We used data from the 2014 and 2016 Korea National Health and Nutrition Examination Surveys. The 2014 dataset was used as a training set, whereas the 2016 dataset was used as the hold-out test set. The synthetic minority oversampling technique (SMOTE) was used to control for class imbalances between the depression and non-depression groups in the 2014 dataset. The least absolute shrinkage and selection operator (LASSO) was used for feature reduction and classifiers in the final model. Data obtained from 9488 participants were used for the machine learning process. The depression group had poorer socioeconomic, health, functional, and biological measures than the non-depression group. From the initial 37 variables, 13 were selected using LASSO. All performance measures were calculated based on the raw 2016 dataset without the SMOTE. The area under the receiver operating characteristic curve and overall accuracy in the hold-out test set were 0.903 and 0.828, respectively. Perceived stress had the strongest influence on the classifying model for depression. LASSO can be practically applied for depression screening of community dwellers with a few variables. Future studies are needed to develop a more efficient and accurate classification model for depression.

## 1. Introduction

Depression causes emotional, cognitive, vegetative, and somatic symptoms, which lead to functional impairment in everyday activities [1]. The prevalence of depression is as high as 10.8% worldwide [2], and it is the single most significant contributor to non-fatal health loss globally [3].

Thus far, increasing evidence indicates that genetic [4], neurogenetic [5], biological [6], and environmental [7] factors contribute to depression. In particular, biological factors such as the level of pro-inflammatory cytokines and brain-derived neurotrophic factors have long been investigated in the field of depression [8,9,10]. However, the presence of such risk factors does not necessarily lead to the future onset of depression. Predictive models capable of indicating who may or may not develop depression are needed. With an emphasis on the practical usefulness of such models in real-world practice, individual-level analyses—rather than group-level analyses—are increasingly important in the field of medicine [11]. Owing to its practical utility, machine learning has received a substantial amount of attention in the field of medicine, including psychiatry [12].

Treatment of individuals with depression is often unsatisfactory. For example, the Sequenced Treatment Alternatives to Relieve Depression (STAR*D) study showed that only one third of the total sample entered remission following initial treatment. In that study, less than 30% of patients achieved remission throughout four consecutive therapeutic regimens [13]. The STAR*D study is not the only clinical study of antidepressants for depression; however, given its large scale and longitudinal style, the influence of the STAR*D study continues to this day [14,15,16]. Therefore, it is necessary to intervene before the onset of a depressive disorder. If we can identify who is more likely to suffer from depression in the near term, we can more effectively prevent depression by focusing on those most at risk.

However, most studies have focused on diagnosing and predicting the prognosis of depression in clinical samples [17,18]. In addition, studies with neuroimaging modalities, such as MRI, largely feature an extremely small sample size, typically less than 100 [18].

Some studies have investigated depression in non-clinical samples using modalities other than machine learning. For example, social media has been widely used, particularly in non-clinical adolescents and youths [19,20,21]. These studies reported that social media usage patterns could meaningfully predict the severity or onset of depression. However, social media can overrepresent young people’s characteristics. As the age at onset of depression extends from adolescence into the early 40s, across almost all sociocultural contexts [22], solely investigating data from social media would limit its applicability to all age groups.

Recent reviews have suggested that machine learning-based approaches have shown some promise in the diagnosis and treatment of depression [17,18,23]. One of the most promising aspects of machine learning is that it provides individual-level results, rather than group-level estimation, of the risk for depression and/or response to treatment. However, many of the machine learning studies that were included in the above reviews suffer from small sample sizes and a lack of separate test sets. These shortcomings can increase the potential risk of overfitting. In addition, the usefulness of focusing on the clinical sample could be limited by the low treatment response rate, as proven by the STAR*D study.

In the present study, we built a predictive model for depression using a machine learning algorithm based on national survey data. Moreover, we identified which variables were the most important for predicting depression.

## 2. Materials and Methods

### 2.1. Participants and Data

The Korea National Health and Nutrition Examination Survey (KNHANES) is an annual nationwide survey that collects a variety of data on health behaviors, the prevalence of chronic diseases, and food and nutrition status. A detailed description of the KNHANES can be found in Kweon et al. [24]. According to guidelines established by the Korean Centers for Disease Control and Prevention (KCDC), depression has been measured biannually since 2014 [25]. We used data from 2014 (*n* = 7550) and 2016 (*n* = 8150).

Only participants who responded to questions that focused on depression and its predictive factors were included in this study. All participants received a full explanation of the aims and protocol of the KNHANES and provided written informed consent. All data processing procedures were approved by the Institutional Review Board of the KCDC (2013-12EXP-03-5C).

### 2.2. Depression and Other Variables

The nine-item version of the Patient Health Questionnaire (PHQ-9) was used to measure depression [26]. As suggested by the KCDC [27], the presence of depression was defined as a score of 10 or higher on the PHQ-9.

Other variables included sociodemographic characteristics (e.g., age, sex, marital status, family income, basic living allowance, and private medical insurance), health (e.g., the prevalence of chronic diseases such as hypertension, diabetes mellitus, and arthritis), quality of life (EuroQol EQ-5D), and laboratory findings (e.g., hemoglobin, hematocrit, white blood cell count, platelet count, blood urea nitrogen level, and urine specific gravity).

### 2.3. Data Preprocessing and Machine Learning

All machine learning processes were conducted using the scikit-learn library implemented in Python 3.7. The 2014 dataset was used as the training and validation sets. Given the unbalanced ratio of depression and non-depression, a synthetic minority oversampling technique (SMOTE) was used [28]. To tune the hyperparameters, 10-fold cross-validation was conducted within the training set. The 2016 dataset was used as a test set to estimate the performance of the classification algorithms built from the 2014 dataset. Categorical variables were converted to dummy variables, whereas continuous variables were transformed into z-scores to ensure that they could be fitted into the linear model, such as regularized logistic regression analysis.

Regularizing the logistic regression model attenuated the overfitting and allowed the classifying model to learn from the training data, not just copy it. Both L1 regularization (also called the least absolute shrinkage and selection operator (LASSO)) and L2 regularization (also called ridge regression) provide a practical solution for overfitting. In a linear regression model, $y=\omega_{0}+\lambda \overset{l}{\underset{k=1}{\sum }}\omega_{k}\chi_{k}$, and LASSO uses a regularization term, $\lambda E\left(\omega \right)=\lambda \overset{l}{\underset{k=1}{\sum }}\left|\omega_{k}\right|$ [29]. As the coefficients of weak predictive variables decrease to zero, LASSO can also be practically used as a feature reduction method.

The regularized logistic regression model has low computing costs and easy-to-understand algorithms, contrary to most other machine learning algorithms that have high computing costs with the black box model.

In this study, we first applied LASSO with the initial 37 contributing variables for feature reduction. Subsequently, we re-entered the resultant 13 variables with non-zero coefficients in the final model. The hyperparameter C, which inversely reflects the strength of the regularization parameter *λ*, was set to 0.0076. As we used LASSO, the penalty option was set to “l1.” Other hyperparameters were set to default in the LogisticRegression scikit-learn library.

### 2.4. Performance Metrics

The area under the receiver operating characteristic curve (AUC) was used as the primary performance metric. Generally, an AUC of 0.8 to 0.9 is considered good, and that >0.9 is regarded as excellent [30]. Other performance metrics such as overall accuracy $\left(\frac{True\;positive\;\left(TP\right)+True\;negative\;\left(TN\right)}{Positive+Negative}\right)$, sensitivity $\left(\frac{TP}{TP+False\;negative\;\left(FN\right)}\right)$, specificity $\left(\frac{TN}{False\;positive\;\left(FP\right)+TN}\right)$, precision $\left(\frac{TP}{TP+FP}\right)$, and Matthew’s correlation coefficient (MCC) were also used $\left(\frac{\left(TP\times TN\right)-\left(FP\times FN\right)\;}{\sqrt{\left(TP+FP\right)\left(TP+FN\right)\left(TN+FP\right)\left(TN+FN\right)}}\right)$. The MCC is superior in utilizing all four principal components (*TP*, *TN*, *FP*, and *FN*) of the confusion matrix. As the MCC is a discretized form of Pearson’s correlational analysis, the value can also be interpreted on the basis of Pearson’s correlational coefficient *r* [31]. Hence, the MCC values range from −1 to 1, unlike other performance metrics with a range of 0 to 1. A value of −1 indicates total disagreement between the actual and predicted values, which coincides with 0 for accuracy. The value of 1 in the MCC indicates a complete agreement between the actual and predicted values, corresponding to 1 for accuracy.

## 3. Results

### 3.1. Participants

After excluding missing cases from the initial 37 variables, 4186 of 7550 (55.4%) participants in 2014 and 5302 of 8150 (65.1%) participants in 2016 were included in the machine learning (Table 1). Table 2 shows the differences in the variables between the depression and non-depression groups.

The prevalence of the minority class (i.e., depression) was 6.16% (584 out of 9488) in the total sample, 6.45% (270 out of 4186) in the 2014 dataset, and 5.92% (314 out of 5302) in the 2016 dataset.

The number (%) of the older adults (i.e., age ≥ 65 years) was 2074 (21.86%). There were significantly higher rates of divorce or separated marital status, older age, and females in the depression group than in the non-depression group. The depression group had significantly lower values than the non-depression group in the socioeconomic domain, such as the number of houses, the number of private insurance policies, receiving a basic living allowance, and household income. The depression group also had a significantly higher prevalence of chronic diseases such as hypertension, dyslipidemia, cerebrovascular disease, cardiovascular disease, thyroid disease, diabetes mellitus, and arthritis compared to the non-depression group. Regarding the quality of life, the depression group had lower scores than the non-depression group on all five domains of the EQ-5D.

### 3.2. Classifying Performance

**Figure 1:**
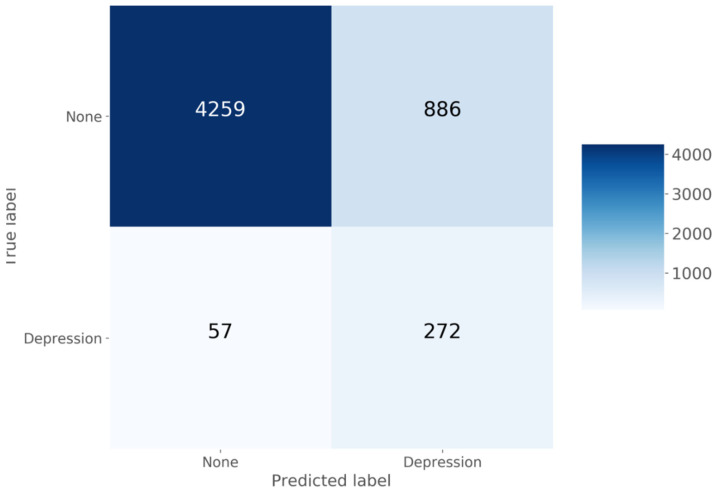
Confusion matrix.

**Figure 2:**
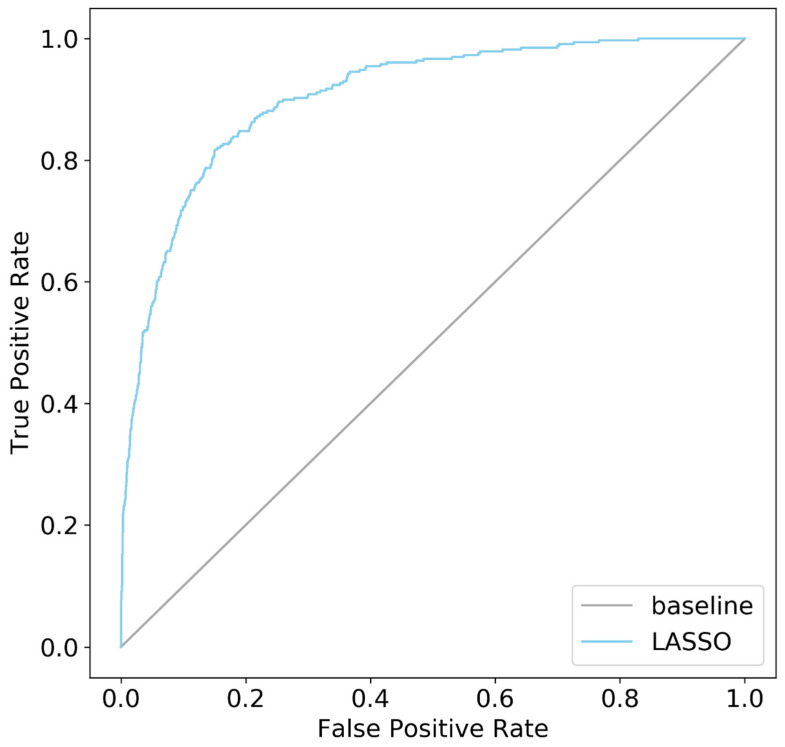
The area under the receiver operating characteristic curve.

As shown in Figure 1 and Figure 2 and Table 3, LASSO showed good classification performance (AUC = 0.903; overall accuracy, sensitivity, and specificity were 0.828). The total number in the confusion matrix of Figure 1 was 5474 because the number of variables was reduced from 37 to 13; accordingly, the number of missing cases decreased. The LASSO model with 13 variables showed a slightly better performance than the model with 37 variables.

### 3.3. Feature Importance

Feature importance was obtained from the magnitude of the coefficients. The variables with the greatest importance were perceived stress, subjective health, anxiety/depression in the EQ-5D, and divorced/separated status (Table 4).

## 4. Discussion

We built a machine learning-based model for predicting future depression. The AUC (0.903), overall accuracy (0.828), sensitivity (0.828), and specificity (0.828) showed that this model could be practically used for screening community-dwelling individuals who may develop depression.

In the final set of variables, perceived stress was the strongest predictor of depression. Stress is generally categorized as either eustress or distress. Eustress represents positive aspects of stress, whereas distress refers to its negative aspects. Perceived stress measures distress by using questions such as “In the last month, how often have you felt nervous and stressed?” The negative effects of stress have a well-documented relationship with the pathophysiology of psychiatric disorders, such as depression [32,33]. As most screening instruments for depression do not contain the term “stress,” perceived stress should be included in screenings of community-dwelling individuals. Moreover, subjective health was ranked as the second most predictive variable for classifying depression. The concept of subjective health reflects the quality of life or well-being [34,35]. Subjective health plays an important role in the pathophysiology of depression [36]. Although depression might contribute to perceived stress and poor subjective health, these factors should be considered important for the early detection of depression.

Our study had several strengths. First, we built a model to classify depression among community dwellers. Although depression causes substantial disability, the treatment of clinical depression is difficult [13]. Hence, early screening and detection of depression among community dwellers are particularly important, and many countries have focused on screening for depression in community settings before the clinical stages of the disease [37,38]. Thus, we believe our model could be practically used in community mental health institutions for accurate and prompt screening of depression.

Second, we used various types of variables. As depression is based on a complex interaction among biopsychosocial variables [39,40,41], clinicians must utilize the possible correlates of depression to improve classification. We included peripheral biomarkers (e.g., thyroid hormone, hemoglobin, white blood cells, platelets, aspartate aminotransferase, and alanine aminotransferase), psychosocial functioning (e.g., EQ-5D), and sociodemographic variables (e.g., age, sex, marital status, educational level, and economic status) to classify depression.

Third, we used LASSO to reduce features and build a final model to classify depression. We found that a model with fewer variables resulted in a performance comparable to one with more variables. We believe that practicality is necessary for such a machine learning model, and from a practical perspective, a questionnaire with too many questions might not be suitable for use in routine screening settings. If the performance between the two models is not substantially different, one with fewer variables could be practically used with the benefits of a short screening time and effort. As we developed this model for use in community health institutions, rather than higher-level facilities, we presumed that low computing costs with fewer variables are an important point. The reasonable computing costs of LASSO facilitate its deployment in community health institutions.

Fourth, it is noteworthy to discuss why we used the 2014 dataset for the training set and the 2016 dataset for the test set, rather than randomly selecting training and test sets. First, we wanted to test whether the algorithm made with past data (i.e., the 2014 dataset) could be applied to future data (i.e., the 2016 dataset). There will be some changes in the frequency or severity of the variables by reflecting the number of times the dataset was collected. If an algorithm should be useful in the real world over time, it should be robust for future data. In addition, there were statistical differences in many of the variables between the 2014 and 2016 datasets, whereas there was no statistical difference in the severity of depression between the two datasets. We interpreted the results mainly in terms of sample size and standard deviation. Generally, as the total sample size increases, the *p*-value decreases [42]. As the sample size was large (*n* = 9488), negligible differences were statistically significant (*p* < 0.05). Moreover, as the standard deviation (i.e., the degree of spread) increases, the *p*-value increases [43]; thus, the non-significant statistical difference in the severity of depression (i.e., PHQ score) resulted from a high standard deviation. As the participants of this study were from the general population, the distribution of the PHQ score would be severely positively skewed, which is associated with a high standard deviation.

This study had several limitations. First, although we included biopsychosocial factors for depression, neuroimaging and genetic variables were not available. Neuroimaging markers, such as structural volumes and functional activity, have long been used to classify depression [44,45]. Genetic studies have also provided information for understanding and classifying depression [4]. As this study sought to create a prompt and accurate tool to classify depression, such expensive tests do not seem applicable for a screening test. Nonetheless, we should consider whether biological factors are, indeed, helpful for discriminating depression. For example, a previous study revealed that the singular use of biomarkers to predict depression prognosis resulted in a poor performance (AUC < 0.6) [46]. The small effects of biological factors were confirmed in our study; only blood urea nitrogen was included in the final model throughout LASSO. Second, due to the limited sample size, we could not subdivide the study population by age group (e.g., youth, middle-aged adults, and older adults); instead, we grouped all ages to build a machine learning model. Given the different contributors to depression across different age groups [47,48], future studies with larger sample sizes are needed. Third, the survey data may not sufficiently reflect respondents’ interpersonal relationships. For example, a recent study revealed that Facebook entries predicted future clinical depression [49]. Although the sample size was small (*n* = 683), and the outcome measure was only moderately predictive (AUC = 0.69 to 0.72), such an approach should be used to supplement future surveys and help construct a more comprehensive dataset.

In summary, we successfully built a model for classifying depression using the LASSO algorithm and sociodemographic, psychosocial, and laboratory data obtained from community dwellers. We believe that this model may help improve the accuracy of depression screening among community-dwelling individuals.

## Figures and Tables

**Table 1:** Sociodemographic, economic, and clinical variables by year.

Variables	2014 (*n* = 4186)	2016 (*n* = 5302)	t or χ^2^	*p*-Value
Age	50.50 (16.03)	50.81 (16.41)	−0.91	0.365
Sex, male	1827 (43.65)	2333 (44.00)	0.121	0.728
Education level			15.108	0.002
Elementary school	903 (21.57)	1091 (20.58)		
Middle school	477 (11.40)	552 (10.41)		
High school	1416 (33.83)	1697 (32.01)		
≥College	1390 (33.21)	1962 (37.00)		
Marital status			6.517	0.089
Unmarried	627 (14.98)	807 (15.22)		
Married	3085 (73.70)	3807 (71.80)		
Widow	174 (4.16)	248 (4.68)		
Divorced or separated	300 (7.17)	440 (8.30)		
Possessing house			0.574	0.751
None	1283 (30.65)	1587 (29.93)		
1	2310 (55.18)	2958 (55.79)		
≥2	593 (14.17)	757 (14.28)		
Basic living allowance, yes	242 (6.26)	336 (6.34)	0.024	0.876
House income, five grades			6.704	0.152
1	787 (18.80)	1105 (20.84)		
2	827 (19.76)	1021 (19.26)		
3	855 (20.43)	1039 (19.60)		
4	836 (19.97)	1018 (19.20)		
5	881 (21.05)	1119 (21.11)		
Private health insurance, yes	3162 (75.54)	4145 (78.18)	9.213	0.002
Hypertension, yes	869 (20.76)	1299 (24.50)	18.56	<0.001
Dyslipidemia, yes	589 (14.07)	899 (16.96)	14.725	<0.001
Cerebrovascular disease, yes	95 (2.27)	109 (2.06)	0.508	0.476
Cardiovascular disease, yes	96 (2.29)	157 (2.96)	4.019	0.045
Arthritis, yes	1018 (11.43)	145 (24.83)	91.44	<0.001
Diabetes mellitus, yes	339 (8.10)	525 (9.90)	9.19	0.002
Thyroid disease, yes	143 (3.42)	211 (3.98)	2.067	0.150
Subjective health	2.87 (0.84)	2.89 (0.86)	−1.206	0.228
Limited activity, yes	285 (6.81)	444 (8.37)	8.085	0.004
Diseased recent 1 month, yes	367 (8.77)	387 (7.30)	6.893	0.009
Aerobic exercise, yes	2263 (54.06)	2407 (45.40)	70.240	<0.001
EQ-5D				
Mobility	1.14 (0.37)	1.15 (0.37)	−0.318	0.751
Self-care	1.03 (0.19)	1.04 (0.20)	−1.661	0.097
Daily activities	1.08 (0.29)	1.08 (0.29)	0.262	0.793
Pain	1.25 (0.48)	1.24 (0.48)	1.004	0.315
Anxiety/depression	1.12 (0.36)	1.11(0.33)	2.167	0.030
Perceived stress	2.91 (0.73)	2.85 (0.74)	3.744	<0.001
Waist circumference (cm)	81.08 (9.75)	82.94 (10.07)	−9.053	<0.001
Hemoglobin (g/dL)	14.09 (1.52)	14.01 (1.60)	2.47	0.014
Hematocrit (%)	41.80 (3.89)	43.17 (4.44)	−15.795	<0.001
Platelet (10^3^/mm^3^)	254.51 (58.87)	258.65 (62.06)	−3.300	0.001
Blood urea nitrogen (mmol/L)	14.45 (4.24)	14.52 (4.71)	−0.743	0.458
Urine specific gravity	1.02 (0.01)	1.02 (0.01)	−2.321	0.987
PHQ-9	2.76 (3.73)	2.68 (3.78)	0.992	0.321

PHQ-9: Patient Health Questionnaire 9 items. All data are presented as mean (standard deviation) for continuous variables and as frequency (%) for categorical variables.

**Table 2:** Sociodemographic, economic, and clinical variables by depression.

Variables	Non-Depression (*n* = 8904)	Depression (*n* = 584)	t or χ^2^	*p*-Value
Age	50.57 (16.13)	52.21 (17.77)	−2.360	0.0183
Sex, male	3993 (44.85)	167 (28.60)	58.77	<0.001
Education level			108.51	<0.001
Elementary school	1777 (19,96)	217 (37.16)		
Middle school	962 (10.80)	67 (11.47)		
High school	2946 (33.09)	167 (28.60)		
≥College	3219 (36.15)	133 (22.77)		
Marital status			181.50	<0.001
Unmarried	1317 (14.79)	1317 (20.03)		
Married	6588 (73.99)	304 (52.05)		
Widow	350 (3.93)	72 (12.33)		
Divorced or separated	649 (7.29)	91 (15.58)		
Possessing house			46.99	<0.001
None	2621 (29.44)	249 (42.64)		
1	4991 (56.05)	277 (47.43)		
≥2	1292 (14.51)	58 (9.93)		
Basic living allowance, yes	481 (5.40)	117 (20.03)	198.89	<0.001
House income, five grades			216.70	<0.001
1	1645 (18.47)	247 (42.49)		
2	1732 (19.45)	116 (19.86)		
3	1808 (20.31)	86 (14.73)		
4	1772 (19.90)	82 (14.04)		
5	1947 (21.87)	53 (9.08)		
Private health insurance, yes	6943 (77.98)	364 (62.33)	77.36	<0.001
Hypertension, yes	1992 (22.37)	176 (30.14)	18.75	<0.001
Dyslipidemia, yes	1358 (15.25)	130 (22.26)	20.36	<0.001
Cerebrovascular disease, yes	170 (1.91)	34 (5.82)	39.88	<0.001
Cardiovascular disease, yes	215 (2.41)	38 (6.51)	35.36	<0.001
Arthritis, yes	1018 (11.43)	145 (24.83)	91.44	<0.001
Diabetes mellitus, yes	771 (8.66)	93 (15.92)	34.95	<0.001
Thyroid disease, yes	322 (3.62)	32 (5.48)	5.30	<0.001
Subjective health	2.82 (0.82)	3.78 (0.90)	−27.21	<0.001
Limited activity, yes	528 (5.93)	201 (34.42)	627.06	<0.001
Diseased recent 1 month, yes	589 (6.62)	165 (28.25)	350.78	<0.001
Aerobic exercise, yes	4422 (49.66)	248 (42.47)	11.36	<0.001
EQ-5D				
Mobility	1.12 (0.34)	1.46 (0.59)	−21.59	<0.001
Self-care	1.03 (0.17)	1.16 (0.40)	−15.84	<0.001
Daily activities	1.06 (0.25)	1.35 (0.53)	−23.80	<0.001
Pain	1.21 (0.44)	1.71 (0.70)	−25.05	<0.001
Anxiety/depression	1.08 (0.28)	1.65 (0.64)	−42.33	<0.001
Perceived stress	2.93 (0.70)	2.02 (0.81)	30.20	<0.001
Waist circumference (cm)	82.14 (9.91)	81.88 (10.91)	0.597	0.551
Hemoglobin (g/dL)	14.07 (1.56)	13.72 (1.57)	5.204	<0.001
Hematocrit (%)	42.62 (4.24)	41.64 (4.30)	5.400	<0.001
Platelet (10^3^/mm^3^)	256.22 (60.21)	266.00 (67.18)	−3.773	<0.001
Blood urea nitrogen (mmol/L)	14.53 (4.48)	13.88 (4.84)	3.333	<0.001
Urine specific gravity	1.02 (0.01)	1.02 (0.01)	2.6249	0.009
PHQ-9	1.98 (2.34)	13.89 (3.63)	−110.0	<0.001

PHQ-9: Patient Health Questionnaire 9 items. All data are presented as mean (standard deviation) for continuous variables and as frequency (%) for categorical variables.

**Table 3:** Performance metrics of the LASSO classifying model for depression.

Number of Variables	Sensitivity	Specificity	Accuracy	AUC	Precision	NPV	MCC
37	0.828	0.822	0.822	0.903	0.226	0.987	0.372
13	0.828	0.828	0.828	0.903	0.235	0.987	0.381

AUC: area under the receiver operating characteristic curve; NPV: negative predictive value; MCC: Matthew’s correlation coefficient.

**Table 4:** Coefficients of contributing variables.

	Coefficients
Perceived stress	−0.8507
Subjective health	0.559
Anxiety/depression in EQ-5D	0.4651
Divorced or separated	−0.261
Male	−0.1446
Possessing ≥ two houses	−0.1131
House income	−0.0939
Pain of EQ-5D	0.0915
Private health insurance	−0.0892
Daily activities of EQ-5D	0.0686
Waist circumference	−0.06
Blood urea nitrogen	−0.0529
Age	−0.006

Bias = −0.5220.

## Data Availability

The Korea National Health and Nutrition Examination Survey (KNHANES) is an annual nationwide survey that collects a variety of data on health behaviors, the prevalence of chronic diseases, and food and nutrition status. We used data from 2014 and 2016. Data are available in a publicly accessible repository. The data in this study are available in Kaggle at https://www.kaggle.com/seoeuncho/predicting-depression-in-community-dwellers (accessed on 20 June 2021).
